# A nomogram prediction for the survival of patients with triple negative breast cancer

**DOI:** 10.18632/oncotarget.24964

**Published:** 2018-08-14

**Authors:** Yuxiang Lin, Fangmeng Fu, Songping Lin, Wei Qiu, Wei Zhou, Jinxing Lv, Chuan Wang

**Affiliations:** ^1^ Department of Breast Surgery, Affiliated Union Hospital of Fujian Medical University, Fuzhou, Fujian Province 350001, China; ^2^ Department of General Surgery, Affiliated Union Hospital of Fujian Medical University, Fuzhou, Fujian Province 350001, China

**Keywords:** triple negative breast cancer, prognosis, nomogram, predictive accuracy

## Abstract

Triple negative breast cancer (TNBC) is a subtype of breast cancer with poor prognosis. In this study, we aimed to conduct a nomogram to predict the survival of individual with TNBC by incorporating significant clinical and laboratory parameters. 404 TNBC patients from the Affiliated Union Hospital of Fujian Medical University between 2006 and 2012 were selected in the training cohort. Cox univariate and multivariate regression analyses were adopted to identify independent prognostic factors. The predictive accuracy and discriminative ability of this nomogram were evaluated by concordance index (C-index) and calibration curve. The accuracy of this nomogram was also compared with the 8^th^ AJCC TNM staging system. An external validation cohort was further performed in an independent cohort of 200 patients between 2012 and 2014. Seven independent prognostic factors, including family history of breast cancer, tumor location, number of positive lymph nodes, histological grade, serum CEA, CA125 and CA153 were identified as independent prognostic factors. A nomogram incorporating these prognostic factors was subsequently conducted and the calibration plot on the probability for 3 or 5 years overall survival (OS) showed an optimal agreement between the nomogram prediction and actual observations. In addition, the C-index of this nomogram was higher than that of TNM staging system in both training and validation cohort (training cohort, 0.76 *vs.* 0.66, *p*<0.001 and validation cohort, 0.72 *vs.* 0.64, *p*=0.002, respectively). This proposed nomogram could provide more accurate individual prediction for the prognosis of the patients with TNBC and was able to help physicians to identify subgroups of patients at different risk and to decide who need intensive follow-up or additional treatment.

## INTRODUCTION

Breast cancer (BC) remains one of the most common diagnosed malignancies and the first leading cause of death from cancer in women worldwide [1]. For the year of 2017, it is estimated in the United States that approximately 252,710 female patients would be diagnosed with BC and 40,610 would die from it [2]. It has been well established that breast cancer is a heterogeneous disease in which the gene-expression profiles vary between individuals [3, 4]. Triple negative breast cancer (TNBC) is a subtype of breast cancer which lacks the expressions of estrogen receptor (ER), progesterone receptor (PR) and human epidermal growth factor receptor-2 (HER2). Although the incidence of TNBC only accounts for a small proportion (10–20%) of all BC [5], it has the worst prognosis and mortality risk than other types of breast cancer in the first three and five years [6–8]. However, for the the absence of ER, PR and HER2 expression, only a few adjuvant treatments, like conventional surgery, chemotherapy and radiotherapy, could provide benefits for patients with TNBC. Therefore, it is important to develop an accurate and practical prognostic predictive model to assist both short-term and long-term treatment decisions for TNBC patients.

Tumor staging system is frequently used to predict the prognosis of patients. The most commonly used staging system for breast cancer is the eighth edition of American Joint Committee on Cancer (8^th^ AJCC) TNM classification [9]. The 8^th^ TNM classification stratifies the patients according to the extent of tumor size (T stage), number of positive lymph nodes (N stage) and distant metastasis (M stage). Although staging system such as the AJCC system is helpful, it is more applicable to a general population rather than individual patients, with the survival of TNBC patients in the same stage may still vary greatly.

Currently, nomograms have been proposed as a reliable tool to quantify risk by incorporating and illustrating important factors for tumor prognosis [10]. Through incorporating significant factors, nomograms could provide a numerical probability of a clinical event, such as overall survival (OS), which is tailored to the profile of individual patient. In several types of cancers, such as intrahepatic cholangiocarcinoma [11–13], hepatocellular carcinoma [14], non-small-cell lung cancer [15] and gastric cancer [16], nomograms have been demonstrated to be more precise when compared with the traditional TNM staging systems. To the best of our knowledge, only one previous report by Dai et al [17] established a nomogram to predict the long-term survival for triple negative breast cancer patients, while this nomogram only involves 247 patients and mainly focused on the value of pre-therapeutic CEA and CA15-3 levels. In addition to this, the study also did not compare the nomogram with the traditional TNM staging systems. Therefore, the present study consisting of 604 TNBC patients was conducted, aiming to develop a simple and practical nomogram for patients with TNBC and compare the performance of this model with the currently available staging system.

## RESULTS

### Characteristic of the study subject

A total of 486 patients diagnosed with TNBC between August 2006 and July 2012 in Affiliated Union Hospital of Fujian Medical University were retrospectively enrolled in this study. The patients who had received prior radiotherapy or chemotherapy (n=58) and lost to follow up (n=24) were excluded. 404 TNBC patients were finally selected in the training cohort. For validation cohort, we enrolled 283 patients and a total of 200 patients between August 2012 and July 2014 were selected according to our inclusive and exclusive criteria. The baseline and clinicopathologic characteristics of the patients in training and validation cohort were listed in Table 1. For patients in the training cohort, the median age at diagnosis was 49.8 years (range, 29 to 65 years). The median OS was 54.6 months (range 4.0 to 131.2 months), and the 1-, 3-, and 5-year OS rates were 94.3%, 74.2% and 60.7%.

**Table 1:** Demographic and clinicopathologic features in the training and validation cohort of patients with triple negative breast cancer

Characteristics	Training cohort (n=404) no. (%)	Validation cohort (n=200) no. (%)
Age, y (mean ± SD)	49.8 ± 10.8	51.1 ± 10.9
BMI, kg/m^2^ (mean ± SD)	22.6 ± 2.8	22.8 ± 3.1
Tumor size, cm (mean ± SD)	2.6 ± 1.3	2.5 ± 1.2
No. of positive lymph nodes (mean ± SD)	1.5 ± 3.7	1.3 ± 3.4
No. of examined lymph nodes (mean ± SD)	20.6 ± 6.2	18.5 ± 7.9
Age at menarche		
≦16	329 (81.4)	157 (78.5)
>16	75 (18.6)	43 (21.5)
Menopausal status		
Premenopausal	222 (55.0)	99 (49.5)
Postmenopausal	178 (44.0)	95 (47.5)
Unnatural menopause^a^	4 (1.0)	6 (3.0)
Age at first live birth		
≦25	237 (58.7)	116 (58.0)
>25	141 (34.9)	60 (30.0)
Nulliparas	26 (6.4)	24 (12.0)
Family history of breast cancer		
No	367 (90.8)	186 (93.0)
Yes	37 (9.2)	14 (7.0)
No. of abortions		
≦1	310 (76.7)	141 (70.5)
>1	94 (23.3)	59 (29.5)
Tumor location		
Upper-outer quadrant	226 (55.9)	98 (49.0)
Lower-outer quadrant	61 (15.1)	34 (17.0)
Lower-inner quadrant	23 (5.7)	8 (4.0)
Upper-inner quadrant	71 (17.6)	52 (26.0)
Undefined	23 (5.7)	8 (4.0)
Histopathological type		
Invasive ductal carcinoma	353 (87.4)	167 (83.5)
Invasive lobular carcinoma	5 (1.2)	1 (0.5)
Others	46 (11.4)	32 (16.0)
Ki67		
≦20%	54 (13.4)	26 (13.0)
>20%,≦40%	118 (29.2)	35 (17.5)
>40%, ≦60%	139 (34.4)	50 (25.0)
>60%	93 (23.0)	89 (44.5)
Grade		
I-II	209 (51.7)	90 (45.0)
III	195 (48.3)	110 (55.0)
CEA (ng/ml)		
≦2.2	289 (71.5)	158 (79.0)
>2.2	115 (28.5)	42 (21.0)
CA125 (U/ml)		
≦17.5	356 (88.1)	156 (78.0)
>17.5	48 (11.9)	44 (22.0)
CA153 (U/ml)		
≦11.3	224 (55.4)	120 (60.0)
>11.3	180 (44.6)	80 (40.0)

^a^ Unnatural menopause includes hysterectomy operation and other status.

### Independent prognostic factors in the training cohort

**Figure 1:**
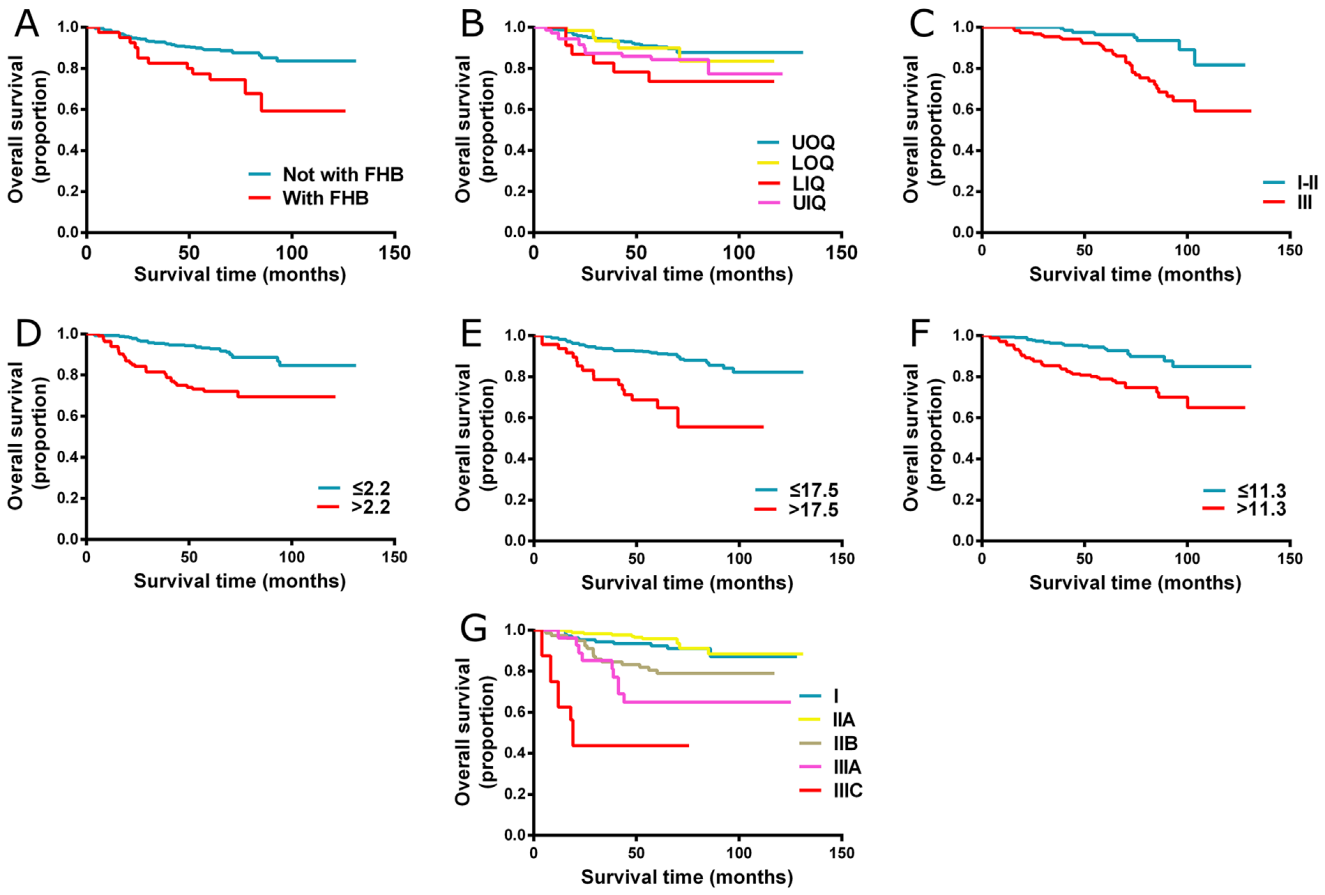
Kaplan-Meier curves for significant factors derived from univariate analysis **(A)** Family history of breast cancer; **(B)** Tumor location; **(C)** Histological grade; **(D)** CEA; **(E)** CA125; **(F)** CA153; **(G)** AJCC 8^th^ TNM staging system] Abbreviations: FHB, family history of breast cancer; UOQ, upper outer quadrant; LOQ, lower outer quadrant; LIQ, lower inner quadrant; UIQ, upper inner quadrant.

We performed the univariate analyses to identify the factors which were correlated with the overall survival of TNBC patients. As shown in Table 2, family history of breast cancer, tumor location, number of positive lymph nodes, histological grade and Ki67 status were significantly associated with the OS. All three laboratory parameters including CEA, CA125 and CA153 were also indicated as significant factors that had impact on survival. The above variables were entered into multivariate Cox proportional hazard regression analyses and the results demonstrated that family history of breast cancer, tumor location, number of positive lymph nodes, histological grade, serum CEA, CA125 and CA153 were independent prognostic factors for OS. The detail results of multivariate analyses are displayed in Table 2. Kaplan-Meier curves for significant factors derived from univariate analysis were shown in Figure 1.

**Table 2:** Univariate and multivariate Cox regression analyses of overall survival for patients with TNBC in the training cohort

Variables	Univariate analyses		Multivariate analyses	
	HR (95%CI)	Log-rank *p*	HR (95%CI)	Log-rank *p*
Factors selected				
Family history of breast cancer	1.61 (1.45-1.83)	<0.001	1.59 (1.41-1.81)	<0.001
Tumor location				
Upper outer quadrant	1.00	0.013	1.00	0.024
Lower outer quadrant	1.02 (0.91-1.17)		1.01 (0.90-1.12)	
Lower inner quadrant	1.31 (1.23-1.52)		1.33 (1.21-1.58)	
Upper inner quadrant	1.24 (1.18-1.48)		1.19 (1.10-1.58)	
No. of positive lymph nodes	1.18 (1.13-1.22)	<0.001	1.21 (1.14-1.28)	<0.001
Grade				
I-II	1.00	<0.001	1.00	<0.001
III	1.50 (1.29-1.68)		1.48 (1.27-1.67)	
CEA (ng/ml)				
≦2.2	1.00	<0.001	1.00	<0.001
>2.2	1.31 (1.18-1.49)		1.28 (1.16-1.51)	
CA125 (U/ml)				
≦17.5	1.00	<0.001	1.00	<0.001
>17.5	1.44 (1.24-1.68)		1.42 (1.21-1.65)	
CA153 (U/ml)				
≦11.3	1.00	0.002	1.00	0.003
>11.3	1.26 (1.10-1.45)		1.24 (1.11-1.44)	
Factors not selected				
Age	1.00 (0.98-1.03)	0.789		
BMI	0.97 (0.88-1.07)	0.576		
Age at menarche	1.13 (0.97-1.32)	0.111		
Menopausal status	0.72 (0.43-1.23)	0.231		
Age at first live birth	0.96 (0.89-1.04)	0.348		
No. of abortions ( >1 vs. ≦1)	0.82 (0.48-1.39)	0.454		
Tumor size	1.13 (0.97-1.33)	0.111		
No. of examined lymph nodes	0.99 (0.95-1.03)	0.547		
Histopathological type	0.78 (0.68-1.09)	0.301		
Ki67 status	1.32 (1.12-1.62)	0.007		

Abbreviations: CEA, carcinoembryonic antigen; CA125, cancer antigen 125; CA153, cancer antigen 153; HR, hazard ratio; CI, confidence interval.

### Prognostic nomogram for OS

**Figure 2:**
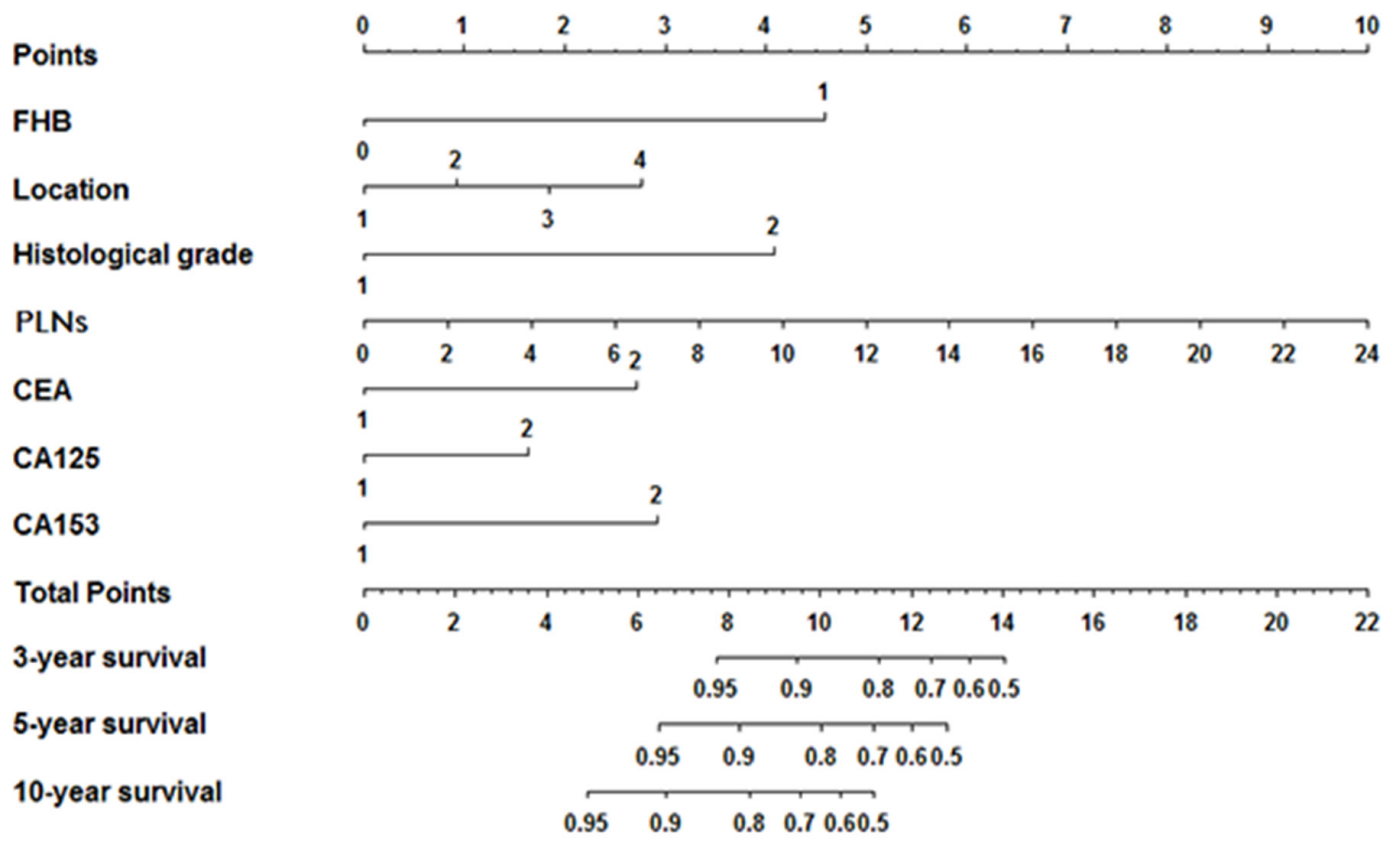
Prognostic nomogram for patients with triple negative breast cancer Abbreviations: FHB, family history of breast cancer; PLNs, number of positive lymph nodes.

**Figure 3:**
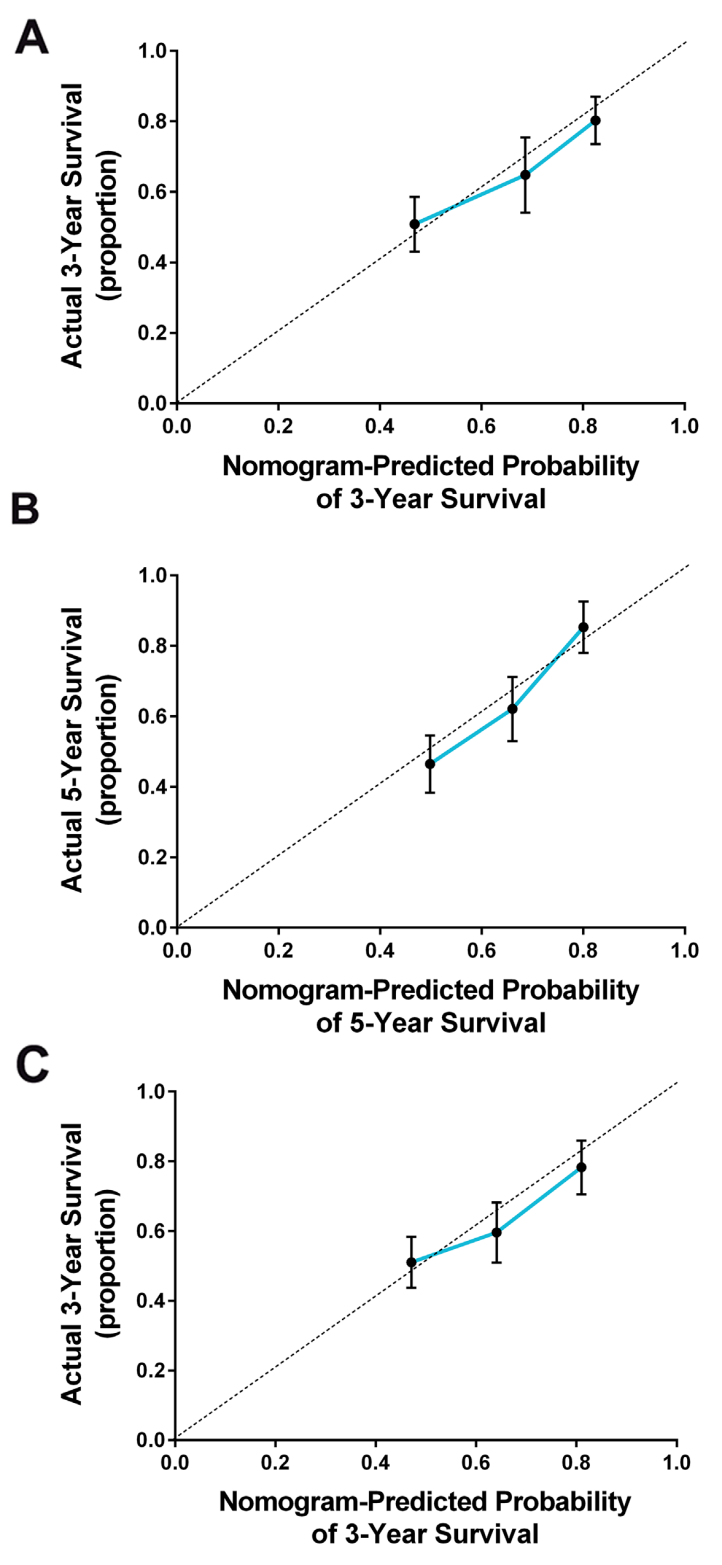
The calibration curve for predicting the overall survival for triple negative breast cancer patients at **(A)** 3 years and **(B)** 5 years in the training cohort and at **(C)** 3 years in the validation cohort. Nomogram-predicted probability of overall survival is plotted on the *x*-axis and the actual overall survival is plotted on the *y*-axis.

All the significant factors identified by the Cox regression model were applied to establish the nomogram for OS. Each subtype within the variables was assigned a score on the point scale. By adding up the total score from all the variables and locating it into the total point scale, we could identify the probabilities of the outcomes by drawing a vertical line to the total score. As shown in Figure 2, the nomogram revealed that FHB (family history of breast cancer) had the largest contribution to the prognosis, followed by the histological grade and serum CA125 level. The C-index calculated in the training cohort for OS prediction was 0.76 (95%CI, 0.72-0.81), indicating the suitability of this new model for TNBC patients. The calibration plot on the probability of survival for 3 or 5 years OS showed an optimal agreement between the prediction by the nomogram and actual observations (Figure 3A and 3B).

### Comparison of predictive accuracy between nomogram and conventional staging system

We also compared the predictive accuracy between this nomogram and the 8^th^ TNM staging system. As shown in Table 3, the performance of nomogram discrimination in the training cohort was 0.76 (95%CI, 0.72-0.81), which was significantly higher than the 8^th^ TNM classification (0.66; 95%CI, 0.63-0.70, *p*<0.001), suggesting better accuracy in predicting OS than the conventional staging system.

**Table 3:** Comparison of the nomogram with TNM staging system

Models	Training cohort		Validation cohort	
	C-index (95%CI)	*p*	C-index (95%CI)	*p*
Nomogram	0.76 (0.72-0.81)	<0.001	0.72 (0.69-0.76)	0.002
8th TNM staging system	0.66 (0.63-0.70)		0.64 (0.60-0.67)	

Abbreviations: C-index, concordance index.

### Validation of the predictive accuracy of the Nomogram for OS

An external validation was further performed in an independent cohort of 200 patients with TNBC. As shown in Figure 3C, the calibration curves also showed good agreement between the nomogram prediction and actual observation for the 3-year OS. In addition, the C-index of the nomogram in the validation cohort for predicting OS was 0.72 (95%CI, 0.69-0.76), which was also superior to the 8th TNM staging system (0.64; 95%CI, 0.60-0.67, *p*=0.002). These results again suggested that this nomogram was useful for predicting the survival of patients with TNBC.

## DISCUSSION

Triple negative breast cancer is a subtype of breast cancer associated with a dismal prognosis [18]. Although the incidence of TNBC only accounts for a small proportion (10–20%) of all breast cancer [5], it has the highest risk of local relapse, distant metastasis and death than other types of BC, especially in the first three and five years [6–8]. TNBC represents an invasive phenotype of breast cancer with diverse molecular characterization and response to therapy [19, 20]. Due to the lack of estrogen receptor (ER), progesterone receptor (PR) and human epidermal growth factor receptor-2 (HER2) expression, only a few adjuvant treatments, like conventional surgery, chemotherapy and radiotherapy, may provide benefits for patients with TNBC. The 8^th^ TNM classification staging system is the most widely used model to predict the clinical outcome of TNBC. However, the prognosis of some patients at same stage may still vary widely. Such heterogeneity might be mainly caused by the different biological and pathological behavior of TNBC. Therefore, it is important to develop an accurate and practical predictive model to assist physicians to make short-term and long-term treatment decisions for specific patients.

Nomogram is a pictorial representation of a complex mathematical formula which uses clinical and biological variables to determine a statistical predictive model and calculates the probability of a clinical event, such as tumor recurrence or death. Nomograms have been proved to be more accurate than the conventional staging systems for predicting the prognosis in several types of cancers [11–16, 21, 22]. In this study, we have developed a novel nomogram by incorporating the clinical and laboratory relevant prognostic factors to better predict the overall survival of TNBC patients. Through univariate analysis and subsequent multivariate regression analyses, we have identified family history of breast cancer, number of positive lymph nodes and histological grade as independent prognostic factors. These findings were in high concordance with previous studies on risk factors for breast cancer [23, 24]. Notably, tumor location was also indicated as an important prognostic factor for TNBC, with which some similar studies also supported that the tumor in lower inner quadrant (LIQ) showed a more unfavorable prognosis [25]. One of the possible hidden reasons is the potential internal mammary node metastasis. The nomogram also includes comprehensive laboratory parameters such as serum tumor biomarkers, which have not been involved in conventional staging systems. As shown in several studies [26–28], higher levels of preoperative serum tumor markers, such as CEA, CA125 and CA153 could represent tumor burden and have been suggested to be independent risk factors for the prognosis of breast cancer. Although these tumor markers have not been included in breast cancer staging systems, their role in increasing predictive performance has been validated in staging systems of other cancer types, such as the hepatocellular carcinoma (HCC) staging systems [29].

To the best of our knowledge, this is the first nomogram for predicting the survival of patients with TNBC which involves both clinical and laboratory relevant factors. The results indicated that this nomogram was superior to the existing staging system with higher C-index and optimal agreement between prognostic prediction and actual observation. Both physicians and patients could perform an individualized survival prediction through this scoring system. This nomogram could help physicians to identify subgroups of patients at different risk of poor survival and to decide who need intensive follow-up or additional treatment. However, several limitations in the current study should also be mentioned. Firstly, the nomogram was established based on the retrospective data obtained from only one single institution in China, although eligibility criteria were formulated to minimize the selective bias. Moreover, our nomogram failed to incorporate some recognized prognostic parameters (eg, vascular carcinoma embolus invasion) or important molecular factors (eg, P53 expression, BRCA1/2 mutation). In addition, relevant treatment conditions such as adjuvant chemotherapy or radiotherapy were also not included in this study. Further efforts on prospective multicenter data collection and incorporation of some other factors are also warranted to improve this model.

In conclusion, this proposed nomogram could provide accurate prediction for the prognosis of the patients with TNBC and was able to stratify patients into distinct prognostic groups. Further perspective cohort studies with larger sample size are also required to illustrate and improve the validity of this model in the therapeutic decision-making field for other ethnic patients with TNBC.

## MATERIALS AND METHODS

### Ethics statement

The study and consent procedure was approved by the Ethics Committee of Affiliated Union Hospital of Fujian Medical University (Fuzhou, China). All participants enrolled in this study provided their written informed consent.

### Study patients and data collection

We conducted a retrospectively study between August 2006 and July 2012 on a training cohort of 486 patients and a prospectively study between August 2012 and July 2014 on a validation cohort of 283 patients. All patients underwent mastectomy or breast-conserving surgery at the Affiliated Union Hospital of Fujian Medical University. The inclusion criteria were as follows: age 20–65 years; no history of previous anticancer treatment; no history of other malignancies; and histopathologically diagnosed with triple negative breast cancer (ER, PR, and HER2 negative. HER2 evaluation was performed using IHC, it was considered as negative of scores were 0 and 1+. For score was 2+, the HER2 status was confirmed either as positive or negative according to the gene amplification ratio of the fluorescence in situ hybridization (FISH) by current standards. For FISH, the cut-off Her-2/CSP17 ratio was defined as 2.0 according to the ASCO guidelines). The exclusion criteria include tumors of uncertain origins or probable metastatic breast tumors; confirmed no metastasis in the preoperative examinations; lack of necessary information; and patients without follow-up data.

**Figure 4:**
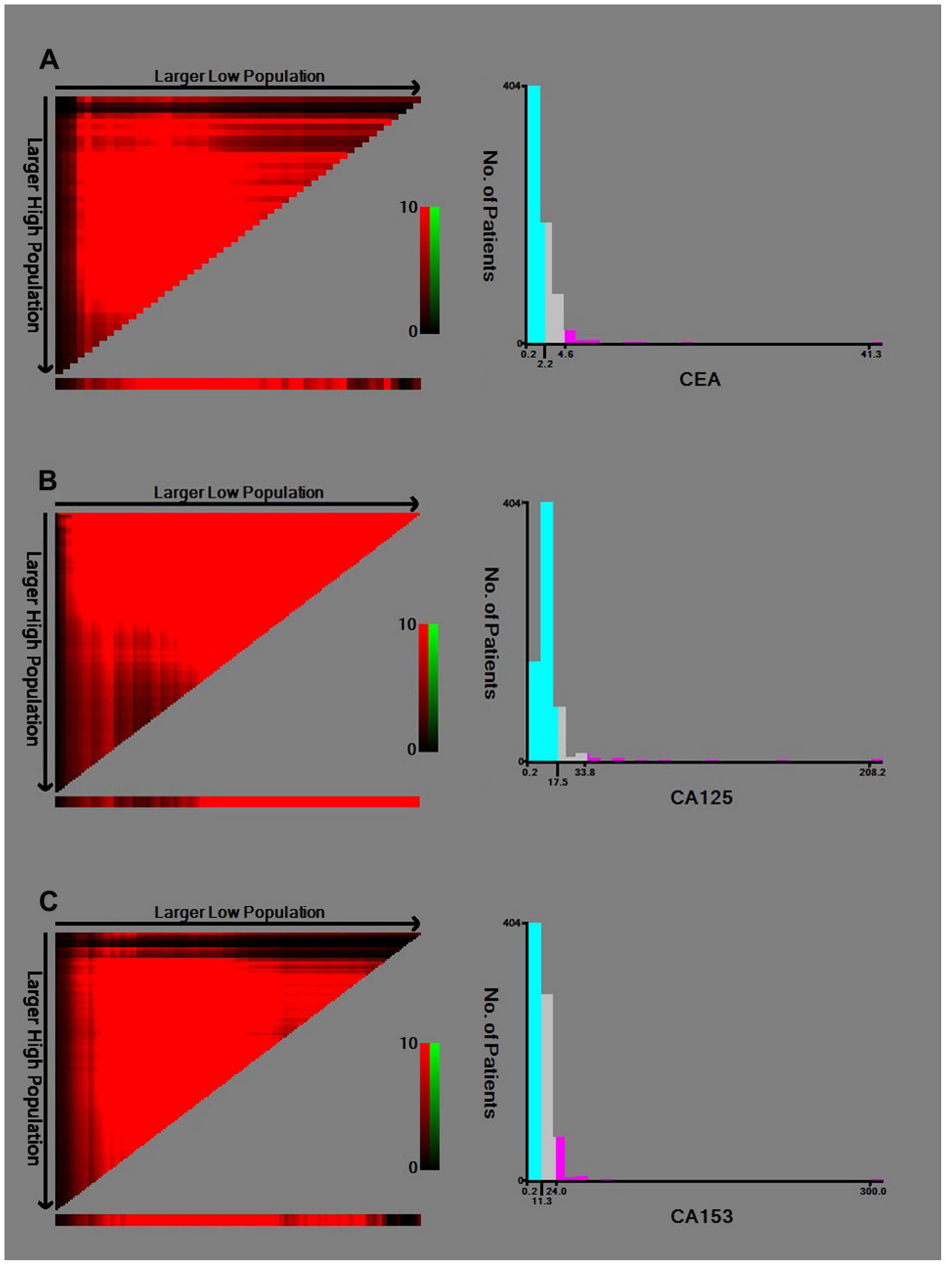
Identification of the optimal cut-off values for serum **(A)** CEA; **(B)** CA125; **(C)** CA153.

Each patient was interviewed face-to-face by a trained interviewer to obtain information on demographic factors (age, BMI, menstrual status, reproductive history, family history of breast cancer). All patients’ clinicopathological data was collected form the electronic medical record systems, including tumor size, lymph node status, tumor location, histopathological type, histological grade, and Ki67 status. The clinical staging of triple negative breast cancer was evaluated by the TNM staging system according to the AJCC 8^th^ edition. Laboratory parameters were retrieved from hematologic tests which were performed at initial diagnosis and prior to any anti-cancer therapy which included carcinoembryonic antigen (CEA), cancer antigen 125 (CA125) and cancer antigen 153 (CA153). The optimal cut-off values for serum cancer biomarkers were determined by the minimum *P* value from log-rank X^2^ statistics using the X-tile 3.6.1 software (Yale University, New Haven, CT, USA) [30]. The cut-off values of CEA, CA125 and CA153 were 2.0 ng/ml, 17.5 U/ml and 11.3 U/ml, respectively (Figure 4).

### Follow-up

All patients were followed-up by telephones calls every 3 months during the first 2 years after treatment, and 6 months annually thereafter. At each follow-up, each patient’s status was carefully recorded. The primary observation endpoint of this study was overall survival (OS) and the last follow-up date was July 1, 2017. OS was defined as the interval between diagnosis and death from any cause or until the last follow-up date which is collected from the patients’ medical records or through direct contact with the patients or their families.

### Statistical analysis

Continuous variables were presented as means ± standard deviation (SD) or medians and ranges, while frequencies and proportions were calculated for categorical variables. The Student’s *t*-test or non-parametric Mann–Whitney U test was performed for continuous data. The Pearson chi-square or Fisher’s exact tests were used to compare the differences in proportion between the groups. Kaplan-Meier method with log-rank test was assessed to depict the survival curves. Cox univariate and multivariate regression analyses were adopted to identify independent risk factors to predict mortality.

Nomogram was built based on the results of multivariate Cox regression analyses using R software (version 3.0.2, http://www.r-project.org/) with the RMS packages. Factors in multiple regression analyses were selected with a backward step-down process, while the smallest Akaike’s information criterion (AIC) was applied as a stopping rule [31]. The performance of the nomogram and TNM staging system for predicting survival were measured by the concordance index (C-index). The maximum C-index value is 1.0 which indicates a perfect prediction model, whereas 0.5 represents only half of the chance to correctly predict the outcome. Bootstraps with 1,000 resample were applied for validation to correct the C-index and explain the variance due to over-optimism. Comparisons between the nomogram and TNM staging system were performed with the rcorrp.cens package of *Hmisc* in R. Calibration curves of the nomogram for 3 year and 5 year overall survival (OS) were adopted to evaluate the agreement between the predicted survival and the observed survival. When externally validating the nomogram, the total points of each patient in the validation cohort were calculated according to the established nomogram, then the total points were used as a factor and applied into the Cox regression model, and finally, the C-index and calibration curve were derived based on the regression analysis. All statistical analyses were two-sided, and a *P* value of less than 0.05 was considered as a significance level.
